# MiRNA-34 and stress response

**DOI:** 10.18632/oncotarget.13923

**Published:** 2016-12-12

**Authors:** Diego Andolina, Di Segni Matteo, Rossella Ventura

**Affiliations:** ^1^ Department of Psychology and Research Center in Neurobiology “Daniel Bovet” Sapienza University, Rome, Italy; Santa Lucia Foundation, Rome, Italy

**Keywords:** miR-34, stress response, anxiety, medial prefrontal cortex, amygdala, Neuroscience

Psychiatric disorders are known to result from a strong interaction between genetic predisposition and environmental factors, mainly exposure to stressful events. Environmental events can modulate genes expression, possibly via epigenetic mechanisms, and affect onset/ expression of a disease [1]. Epigenetic mechanisms include, among others, post-transcriptional regulation by non-coding RNAs such as microRNAs (miRNAs). MiRNAs are small non-coding RNAs predicted to regulate hundreds of targets and to be engaged in every biological process [2]. Thanks to their ability to fine-tune gene expression, miRNAs can control gene expression patterns favoring organism’s adaptation to internal and environmental (external) factors [3], such as stressful events.

Studies in humans and in animal models have provided important insights into the role of miRNAs in different psychiatric disorders, showing that miRNAs are involved in neuroplasticity, neuronal adaptation to stress, and stress-related disorders including anxiety, depression, and bipolar disorder [1, 3-5]. In particular, animal models offer the opportunity to correlate miRNA expression in specific brain structures with different behavioral phenotypes [1, 3-5].

Recent evidence point to members of the miRNA-34 family of miRNAs as a critical modulators of stress response, showing their role in the manifestation of fear and anxiety-related behaviors [6, 7]. Although different brain areas are involved in stress response modulation, the medial preFrontal Cortex (mpFC) and the amygdala are crucially affected by stressful stimuli, and multiple lines evidence indicate that dysfunction of the neural circuit connecting these two structures underlies stress-related anxiety-like disorders [7]. Interestingly, miRNA- 34c (a member of the miR-34 family) has been reported to be up-regulated in the central nucleus of the Amygdala following acute and chronic stress in mice [6]. Moreover, local inhibition of miRNA-34c increased anxiety-like behavior, while its ectopic expression partially reverted this phenotype [6].

We have recently demonstrated that serotonergic prefrontal transmission modulates the stress response acting on GABAergic transmission within the basolateral amygdala (BLA) in mice [8]. Using mice carrying a targeted deletion of miR-34a, miR-34b, and miR-34c (TKO), we have shown that miRNA-34 expression within the prefrontal-amygdala brain circuit regulates stress response. We evaluated the role of miRNA-34 in stress response by subjecting wild type (WT) and TKO mice to different anxiety-related tests (elevated plus maze, dark-light and open field tests). We also investigated prefrontal serotonergic-amygdalar GABAergic release induced by acute restraint stress exposure as well as dendritic remodeling induced by stress in the BLA of WT and TKO mice. We found that TKO mice had a reduced behavioral (anxiety-like behaviors), morphological (reduced spine density, numbers of branch points, dendritic length in amygdala), and neurochemical (prefrontal serotonin/ norepinephrine-amygdalar GABAergic release) response to acute stress exposure, thus suggesting that absence of miRNA-34 protects from stress-induced anxiety-like phenotype expression.

Interestingly, under unstressed conditions WT and TKO animals did not show any significant difference in anxiety-like behaviors, neurotransmission and morphological parameters in the BLA. This is consistent with the view that many miRNAs act as modulators of stress responses and with the observation that many miRNA knockout animals display altered phenotypes only following environmental stress exposure [2].

Although data from knockout models might partially be affected by developmental compensatory modifications, our data point to a modulatory role of miRNA-34 in the response of prefrontal serotonergic-amygdalar GABAergic brain circuit to stress events, in agreement with studies suggesting specific functions of miRNAs in the regulation of brain neurotransmission activity.

Investigating and confirming the interaction between miRNA-34 and its putative target genes will shed light on the mechanisms by which miRNA-34 can regulate different biological processes, including stress response. Among the putative targets, the stress-related corticotropin releasing factor receptor type 1 (CRF1) mRNA is one of the most important target of miRNA-34, and the role of miRNA-34 as regulator of CRF signaling has been recently reported demonstrating that miRNA-34 reduced the responsiveness to CRF of CRFR1-expressing neuronal cells [6]. Because reduced stress effects (and increased “active coping” behavior) have been associated to a inhibition of dorsal raphe nuclei (DRN)-serotonin system projecting to the pFC mediated by endogenous CRF through CRFR1 receptor and we observed a reduced prefrontal serotonergic response to stress in TKO mice, we speculate, also based on preliminary data from our laboratory (data not shown) that “resilience” to stress showed by TKO animals might be due to increased DRN CRF1 receptors expression (induced by miRNA-34 lack), that inhibits serotonin prefrontal stress-induced release thus blunting amygdalar GABAergic outflow and, lastly, promoting a more “resilient” phenotype.

Overall, these data indicate an important role of the miR-34 family in modulating stress responses in the CNS. Further studies investigating whether miR-34-related-single nucleotide polymorphisms can be used as predictive risk indicators for the expression of psychopathologies induced by stress are certainly warranted.

Finally, peripheral circulating miRNAs have been proposed as potential biomarkers of different psychopathologies and useful tools to evaluate treatment response [1, 5]. Despite some promising results in this area, much more has to be done to elucidate the functions of these small non-coding RNAs and to understand their potential role in the molecular pathways altered in many psychopathologies.
